# Analyses of Influence on Chromium Coating after Grinding from the View of Final Microstructure and Microhardness in the Surface Layer

**DOI:** 10.3390/ma14092396

**Published:** 2021-05-04

**Authors:** Nataša Náprstková, Martin Novák, Martin Marek, Karel Šramhauser, Jan Sviantek, Dana Stančeková, Miroslava Ťavodová

**Affiliations:** 1 Faculty of Mechanical Engineering, Jan Evangelista Purkyně University in Ústí nad Labem, Pasteurova 7, 40096 Ústí nad Labem, Czech Republic; martin.novak1@ujep.cz (M.N.); karel.sramhauser@ujep.cz (K.Š.); jan.sviantek@ujep.cz (J.S.); 2 Solar Turbines AEME Ltd., 43108 Bitozeves, Czech Republic; marek@solarturbines.com; 3 Faculty of Mechanical Engineering, University of Zilina, Univerzitná 8215/1, 01026 Žilina, Slovakia; dana.stancekova@fstroj.uniza.sk; 4 Faculty of Technology, Technical University in Zvolen, Študentská 26, 96001 Zvolen, Slovakia; tavodova@tuzvo.sk

**Keywords:** grinding, microstructure, SG, SiC, cutting speed

## Abstract

The article deals with the analysis of chromium layer grinding on a steel substrate, where this issue was addressed with regard to the requirements of the manufacturing sector, specifically in the aerospace industry. The experimental samples were chromium-plated and ground under different cutting conditions by the grooving method of grinding. Two types of grinding wheels for grinding were used, grinding wheel based on SG (solgel) a grinding wheel based on SiC. The resulting microstructure and microhardness in the machined layer were evaluated with using of confocal laser microscopy, inverted materials microscopy, and hardness testing. Based on the results, recommendations were made regarding a suitable approach to grinding the chromium coating. We used a confocal laser microscope and hardness tester for the evaluation of presented values. It was found that, on the base of analyses values, with both grinding wheel and using cutting conditions used, good results have been achieved. This could be stated, because the analysis of the samples microstructure after grinding for the given cutting conditions showed that it is possible that a small influence is completely acceptable from the point of the final product view and there are no major negative phenomena. Measurements of surface microhardness after grinding showed similar results for all samples. The SiC-based grinding wheel showed slightly better results, but both grinding wheels can be used without problems for the presented cutting conditions, and the presented cutting conditions with both grinding wheels can be recommended for the grinding of the given material.

## 1. Introduction

As many sources state, e.g., [1,2,3], the machining of chromium and its coatings, respective its grinding, has some specific complications, because it represents the machining of hard and, therefore, difficult to machine materials, which is a notorious thing, [4,5] or [6]. The requirements for machining such materials are based on the need for engineering production, especially specialized production, such as the aerospace industry, for example.

As sources state, for example [7,8], chrome coating is mainly used as protection against corrosion, erosion, and abrasion, and this is also used when an aesthetic effect is needed, which is also mentioned in [9]. It is often found where it is necessary to renovate worn parts and its function is often also decorative (e.g., tools, bicycles, automotive industry, and sanitary ware), which mentions e.g., [9,10]. Thus, it can be stated and, as they also state, for example [11,12,13,14], chrome surfaces are used to increase the service life of stressed components.

Chromium belongs to the category of difficult-to-machine materials that is applied by galvanic (electro-chemical) plating (chromium plating) and, therefore, it is processing by machining, in our case grinding, is current and the solution of these issues is demanded by industry, as they state e.g., [15,16]. Hard chrome plating technology satisfies the specific requirements of engineering production. However, it should be noted that new surfaces that are created by the application of a layer of hard chromium usually need to be further processed to achieve the desired quality of the resulting surface. The hardness of the applied chromium layer should usually be at least 900 HV (66.5 HRC). The thickness of chromium coatings is usually in the range of 25–500 μm, [15,16]. These surfaces must then be finished by grinding, as the applied layers are not so large as to allow the use of turning or milling.

Because it is a matter of machining thin surfaces, grinding is the only machining method that can be considered here, as mentioned above. Grinding technology occupies an important place among other machining technologies and it is mainly used because the obtained surfaces meet considerable demands on quality parameters and there is a removal of very small chips, so it is possible to machine the coatings [1,3].

The result of grinding, as with other machining technologies, it is of course associated with several factors. For example, Maruda et al. [17] wrote about changes of structure and microhardness changes after AISI 1045 steel turning for minimum quantity cooling lubrication. Alternatively, Nadolny and Kieraś [18] researched the MQL-CCA method of applying coolant during the internal cylindrical grinding process. Khan et al. [19] wrote about research of AISI D2 Steel Grinding with Al_2_O_3_ Wheel under minimum quantity lubrication (MQL).

Various abrasive materials are used to grind more difficult to machine materials, and extensive research is being conducted in this area, which, of course, affects the resulting surface and its quality in various ways, i.e., the surface integrity. For example, Ding et al. [20] wrote about research of monolayer CBN superabrasive wheels application for grinding metallic materials and Wang et al. [21] about influence of electroplated CBN wheel wear on powder metallurgy superalloy FGH96 grinding. The topic of grinding (machining) and influencing the ground material by various parameters are also dealt with by [22], when there was presented research about using low-temperature coolants in machining to enhance the durability of AISI 316L stainless steel or [23], when there was presented research about grinding of titanium alloy Ti6Al4V with silicon carbide grinding wheel. Taylor and Slatter [24], etc., wrote about the role of temperature parameters in achieving precision traverse cylindrical grinding of chrome-plated ferrous metal.

The integrity of the surface, as a complex result of machining (grinding) and an important indicator of machining quality, is formed by many elements and its evaluation is dealt with by many researches, because the integrity of the resulting surface, especially some of its elements, testifies to the quality of the implemented technology, suitably or inappropriately selected cutting conditions, possibilities of used machining tools, etc. Jawahir et al. [25] described surface integrity in material removal processes, or Novák [26] wrote about selected parameters of surface integrity during grinding of hardened steels. Marek et al. [27] and Marek and Novák [28] wrote about impacts of changes in feed rate on selected materials surface integrity. The resultant roughness, as described in [29] or [30], is also a frequently evaluated component of surface integrity. In the presented area, various methods are used to evaluate surface integrity elements. Some methods are mentioned e.g., in [31,32,33].

There is a limited amount of knowledge from the field of grinding the chromium layer with grinding wheels based on SG and SiC, although these wheels are used quite frequently, especially for practical use, and this knowledge is constantly in demand by industry. The information obtained always applies to specific conditions (cutting conditions, workpiece material, grinding wheel material, etc.). The motivation of this research is the expansion of knowledge and information on this question and the availability of a wider range of information for industry.

## 2. Experiment

The presented research is part of long-term activities in the field of grinding research and long-term cooperation of this workplace with industry and manufacturing. Experimental material, used equipment, and grinding conditions (the cutting conditions) are described below.

### 2.1. Experimental Material

The choice of base material for the coating was based on its current use in the industrial sphere. It was chromium-nickel-molybdenum steel marked as AMS6415 according to SAE (Society of Automotive Engineers) or alloyed steel AISI 4340. This material is resistant to embrittlement and it has high strength, toughness, and its mean elongation value is 10%. AISI 4340 steel is used in most industries for applications that require higher tensile strength (for highly stressed components). In Table 1 and Table 2, there is the possibility to see the chemical composition and mechanical properties of the alloy AMS6415 (according to the material lists [34,35].

A spectrometric analysis was performed on an optical emission spectrometer Q4 TASMAN (produced by Bruker-Quantron, Kalkar, Germany) to verify the chemical composition of the samples. Table 3 presents the resulting values of the measurement of the chemical composition of the sample substrate. A comparison of Table 2 and Table 3 shows that the chemical composition according to the material list corresponds to the actual chemical composition of the samples. A chromium layer was galvanically applied to the base material using hard chrome plating technology. In order to verify its chemical composition, an analysis was performed using a hand-held optical spectrometer DELTA (Hørsholm, Denmark), which verified and confirmed the purity of the applied coating (100% Cr).

The thickness of the chrome layer was measured before the grinding. The average thickness of the chromium layer before grinding was 392 μm. It was also necessary to evaluate the porosity in the structure of the uncut sample for subsequent comparison with the ground samples.

### 2.2. Experimental Setup

**Figure 1:**
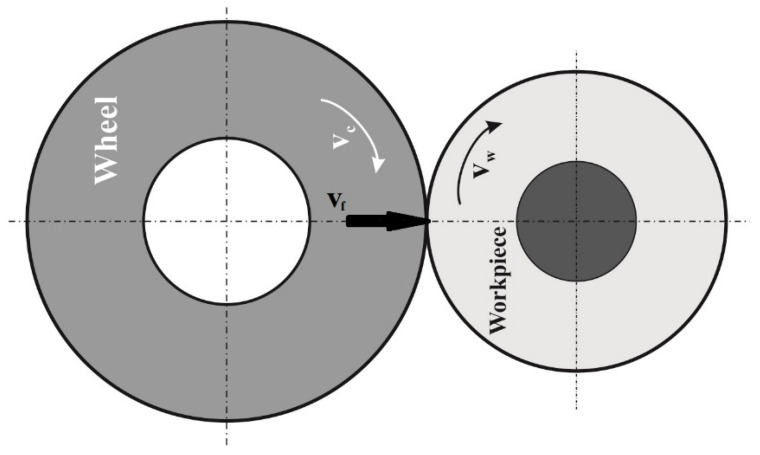
The grinding schema used in the experiment (v_c_—cutting speed, v_f_—radial feed of tool, v_w_—circumferential speed of the workpiece).

The grinding of the samples was performed on a BU16 semiautomatic center grinder (TOS HOLICE, Holice, Czech Republic). A diamond pencil dresser has been used to dress the grinding wheels. In the case of our experiment, the peripheral cylindrical grinding (grooving in this case) method has been used to grind the samples, see Figure 1. The grinding wheel was wider than the ground sample.

In the experiment, two types of grinding wheels were used as grinding tools, where wheels that were suitable for processing hard materials were selected, which, of course, includes chromium.

The first wheel was a grinding wheel with a newly developed microcrystalline corundum-based grains type, called SG. SG production technology is based on controlled crystallization from solid solution in the presence of a catalyst. These grains have a greater ability to remove material, and grinding wheels that are made from this material require fewer dressing cycles. Their main use is in the grinding of hard materials. In terms of hardness, it is a softer disc with very fine grain size, medium porosity, and ceramic binder. The grinding wheel with a diameter of 300 mm allows a maximum cutting speed of 40 m·s^−1^ and it is marked AG 92/99 150 J 9V.

The second experimental wheel was a grinding wheel on the base of silicon carbide (SiC). This grinding wheel is also suitable for machining hard materials. When compared to the previous wheel, it is characterized by a different (more pronounced) breakage of blunt grains and replacement with new, sharper grains. It is a grinding wheel of fine grain, very porous structure for better chip removal from the working environment, and with a ceramic binder. Additionally, like the previous wheel, it is wheel with diameter allows a maximum cutting speed of 40 m·s^−1^, and it is marked C49 150 J 9V.

The determination of cutting conditions was an important part of the experiment. These were the cutting speed of the tool v_c_, the circumferential speed of the workpiece (sample) v_w_, the radial feed of the tool v_f_, the depth of cut a_e_, and the cutting environment.

The cutting speed v_c_ has a limiting factor of the maximum circumferential speed. This value is indicated on each grinding wheel. Both types of selected grinding wheels had, as mentioned above, a maximum allowable peripheral speed of 40 m·s^−1^. The cutting speed for the experiment was chosen in two values, 30 and 40 m·s^−1^. The circumferential speed of the workpiece v_w_ was set to 15 m·min^−1^. This value was chosen based on previous experience, and it is also one of the parameters that were constant throughout the experiment. The radial feed of tool v_f_ was chosen based on the possibilities of the experimental grinder. The removal depth a_e_ was chosen 0.05 mm per cut. Table 4 summarizes all of the experimental values of the selected cutting conditions. The used cutting conditions were determined on the basis of experience from other already performed experiments, e.g., [34,35].

The cutting environment was chosen based on the previously performed experiments of research conducted at FME JEPU in the field of research of process fluid influence on the properties of the resulting ground surface. In this case, it was the process fluid Hakufluid 182, which is suitable for use in precision grinding and other finishing operations.

When compared to the values that were obtained after grinding, a sample that is not ground, was only chrome-plated, was also measured and analyzed, as described above.

## 3. Results and Discussion

After the experiment, the ground samples were analyzed and evaluated. Within the samples, some of the selected components of surface integrity were evaluated. Thus, after grinding, changes in microstructure, cracking, and porosity were assessed, and the course of microhardness in the surface layer after grinding (chromium layer, substrate) was measured and analyzed.

### 3.1. Evaluation of Microstructure

The possible changes in microstructure were one of the monitored components of surface integrity. Because the grinding process material is subjected to thermal and mechanical loads, it can cause changes in the preform microstructure. This change may or may not be beneficial to the nature of the process, so it is worth paying attention to this aspect. The microstructure evaluation was monitored using an Olympus LEXT OLS3000 confocal scanning laser microscope (manufactured by Olympus, Tokyo, Japan). Metallographic samples were prepared after grinding in the usual way to assess these changes. The samples were etched with 4% nitric acid.

Partial microstructure evaluation also estimates the porosity layer of chromium after the grinding, as it may be caused by thermal influence during grinding. The resulting assessment of porosity and possible cracks after grinding is one of the important components of assessing the integrity of the surface, since, as mentioned above, during the grinding process, there is usually high mechanical and thermal stress on the ground parts, which can subsequently affect the crushed material. The penetration of pores in the ground layer may be one of the features of such an effect. Therefore, during the experiment was also evaluated in the percentage of porosity in a layer of chromium. In each sample, five different areas were always measured and averaged over these.

**Figure 2:**
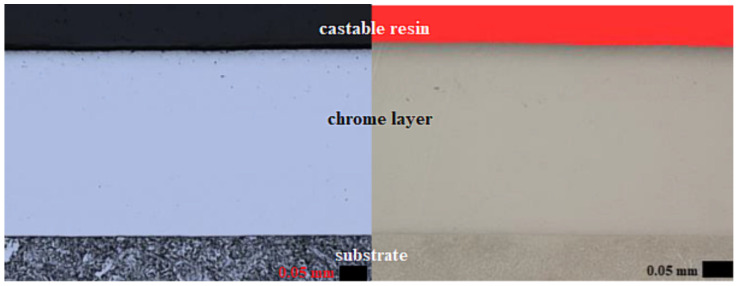
The microstructure and porosity of uncut sample (sample 0).

Porosity analysis was performed by using image analysis of samples that were processed in accordance with Table 4. (Samples A1 to A10 and B1 to B10). To analyze the images, it was necessary that the samples under study were not etched, and the area was scanned immediately after polishing. The only way that you can get a clean unoxidized surface and, thus, obtain a better contrast between themselves and the material pores. Nikon Eclipse Ma200 microscope (manufactured by NIKON company, Minato, Japan) was used to measure the porosity of the material. The captured area has been converted to grayscale. Subsequently, the contrast has been increased due to the linear transformation point image. Subsequently, grayscale images have been converted into a segmented image. Segmented images have only two possible values, 0 for the background and a maximum value of 255 for the objects. The result of thresholding is then a two-color image, where the pores (or cracks) are highlighted in red on the background, see Figure 2.

The chromium layer, transition area, and base material were monitored as part of the microstructure observation. The microstructure observation of these three areas was carried out because the samples could be subjected to excessive mechanical and thermal stress during grinding, which could cause changes in the material structure. The law of preservation of properties, which applies by default during grinding, stipulates that the structure of the material must be preserved before and after grinding, and it must not be changed.

Figure 2 shows the uncut chromium layer, as well as the substrate itself. Additionally, Figure 1 showed the analysis of the porosity image of this sample. In the structure of the chromium layer, there is very little apparent porosity (almost negligible). Moreover, the measurements showed that the mean porosity in the uncut layer of chromium was within 0.03%.

**Figure 3:**
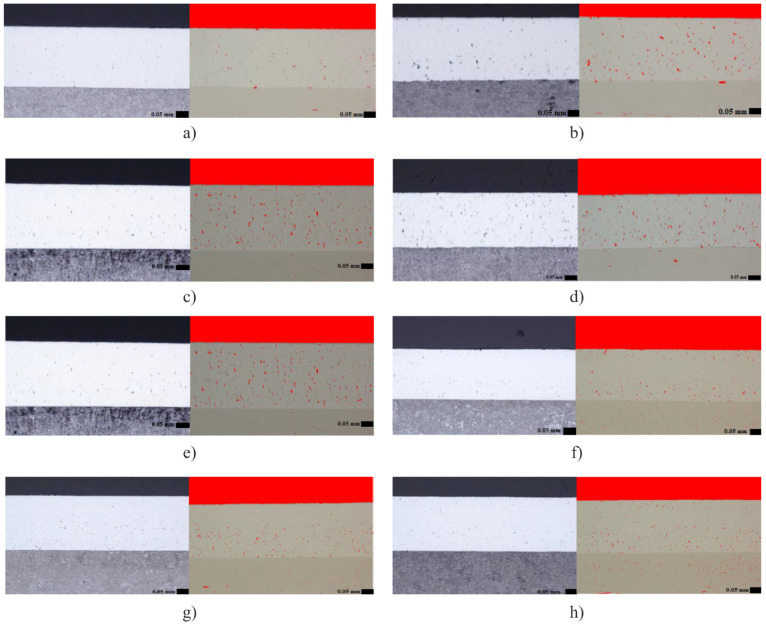
The microstructures and porosity of samples after grinding, (**a**) sample A1 (SG, v_c_ = 30 m·s^−1^, v_f_ = 0.13 mm·min^−1^), (**b**) sample A5 (SG, v_c_ = 30 m·s^−1^, v_f_ = 0.64 mm·min^−1^), (**c**) sample A6 (SG, v_c_ = 40 m·s^−1^, v_f_ = 0.13 mm·min^−1^), (**d**) sample A10 (SG, v_c_ = 40 m·s^−1^, v_f_ = 0.64 mm·min^−1^), (**e**) sample B1 (SiC, v_c_ = 30 m·s^−1^, v_f_ = 0.13 mm·min^−1^), (**f**) sample B5 (SiC, v_c_ = 30 m·s^−1^, v_f_ = 0.64 mm·min^−1^), (**g**) sample B6 (SiC, v_c_ = 40 m·s^−1^, v_f_ = 0.13 mm·min^−1^), (**h**) sample B10 (SiC, v_c_ = 40 m·s^−1^, v_f_ = 0.64 mm·min^−1^).

Figure 3 shows obtained microstructures and image analyses of porosity of individual samples ((a) A1, (b) A5, (c) A6, (d) A10, (e) B1, (f) B5, (g) B6, (h) B10) after grinding, always for the smallest and largest v_f_ within the SG and SiC wheel and v_c_ = 30 m·s^−1^ and v_c_ = 40 m·s^−1^, also see Table 4.

For v_c_ = 30 m·s^−1^ (SG, samples A1 to A5, Figure 3a,b), with increasing v_f_, it was possible to observe that there was a percentage increase in porosity. The lowest porosity value was achieved in sample A1, where the porosity was 0.31 ± 0.10%, and the highest value was reached in sample A5, where the value climbed to 2.15 ± 0.22% porosity. For the lowest feed rate v_f_ = 0.13 mm·min^−1^ (sample A1, Figure 3a) pore size of approx. 10 μm was measured. For the highest feed rate v_f_ = 0.64 mm·min^−1^ (sample A5, Figure 3b), the pores reached dimensions of approx. 22 μm. As expected, the microstructure of the base material did not change.

Figure 3c,d shows the obtained microstructures and image analyses of porosity of individual samples (Figure 3c A6, Figure 3d A10) after grinding, always for the smallest and largest v_f_ within the SG wheel and v_c_ = 40 m·s^−1^.

For v_c_ = 40 m·s^−1^ (SG, samples A6 to A10), it was possible to observe that the porosity values also increased (albeit slightly) due to increasing v_f_. The smallest value was reached in sample A6, where the porosity was measured at 1.51 ± 0.19%, and the highest value was reached in sample A10, where the porosity was measured at 2.60 ± 0.26%. As in the previous case, according to the assumption, the microstructure of the basic material did not change.

Similar results could be observed for samples that were machined with the SiC wheel.

Figure 3e,f show the obtained microstructures and image analyses of porosity of individual samples after grinding (B1, B5), always for the smallest and largest v_f_ within a SiC wheel and v_c_ = 30 m·s^−1^.

For v_c_ = 30 m·s^−1^ (samples B1 to B5) with increasing feed rate, a percentage increase in porosity could also be observed. The lowest value of porosity was achieved in sample B1, when the porosity was around 0.58%, and the highest value was reached in sample B5, where this value reached 1.61 ± 0.16% of porosity. The size of the pores was between values 12 to 22 μm.

When the cutting speed was increased to v_c_ = 40 m·s^−1^, the resulting porosity values did not have an increasing tendency in connection with the increase of the feed rate.

**Figure 4:**
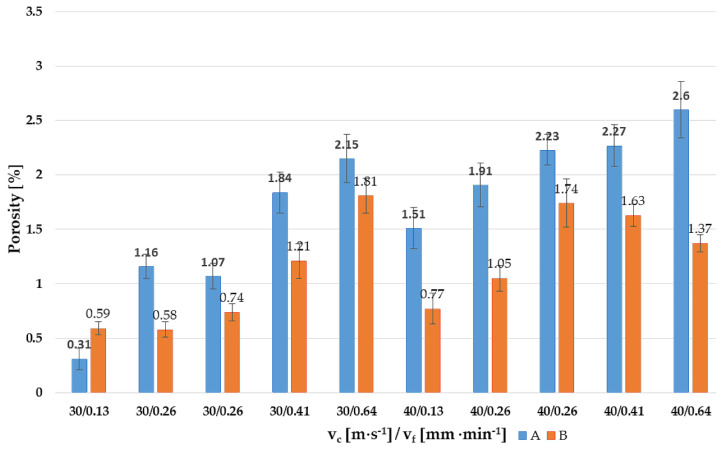
The porosity in grounded samples (A1 to A10, SG; B1 to B10, SiC).

When the cutting speed was increased to v_c_ = 40 m·s^−1^, the resulting porosity values did not have an increasing tendency in connection with the increase of the feed rate. For sample B6, the porosity value was 1.74 ± 0.22%. Subsequently, the samples showed decreasing porosity values for each vf, with sample B10 showing a porosity of 1.37 ± 0.08%. The size of the maximum visible pores ranged up to a maximum of 30 μm. Figure 4 shows these summarized presented facts in the graph.

### 3.2. Evaluation of Microhardness in the Surface Layer

Another part of the experimental analysis was to perform microhardness measurements in the surface layer of the ground sample to determine how the cutting conditions affect the final surface layer hardness, which can often be observed for machined surfaces [22,23,24,25]. The hardness tester LECO LM 248AT (manufactured by the LECO company, St. Joseph, MO, USA) was used to measure microhardness. The magnitude of the load when analyzing the microhardness was selected 0.98 N per 10 s (HV0.1).

Each sample was cut at an angle of 3° to a depth of 0.6 mm. The course of microhardness in the surface layer and substrate was determined by the successive making of test impressions in a row. Thus, three series were measured for each sample, from which it was determined the average rate of this value.

From the point of view of internal regulations of qualified suppliers for chromium plating, the values of microhardness in the chromium layer should be around 950 HV0.1 ± 3 to 5% and the values of microhardness of the base material around 340 to 360 HV0.1.

The experiment evaluated the influence of cutting conditions, especially the change in grinding wheel speed, grinding wheel feed rate, and grinding wheel materials on this quantity. The number of pinholes varied as the chrome layer always needs to be ground to a circle. The sample was fully engaged, so the resulting chromium layer thicknesses after grinding were different. As a result of this fact, in the chromium layer were performed three to five injections, eight to ten injections were made in the base material (substrate), and a total of three series of measurements were always performed. The measurement always started at 0.05 mm from the edge of the sample (the beginning of the chromium layer), and punctures were always made every 0.05 mm to a length in the range of 0.2 mm (respectively, 0.25 mm). In the substrate, the measurement always started 0.25 mm (respective 0.3 mm) from the edge of the sample (including the chromium layer) to a distance of 0.65 mm (respectively, 0.7 mm) in increment 0.05 mm.

**Figure 5:**
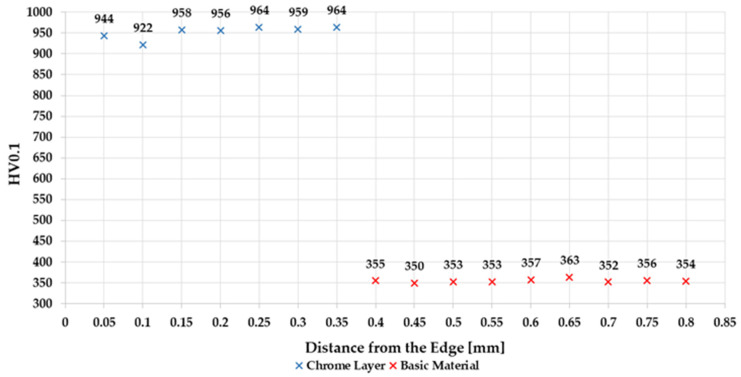
The course of microhardness in sample 0.

Figure 5 shows the course of microhardness in the chromium layer and in the base material of the unground sample. The measured values correspond to expectations.

**Figure 6:**
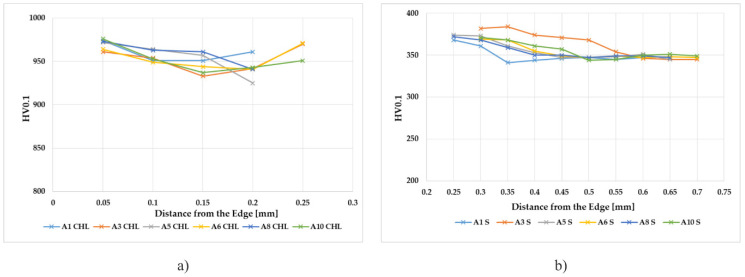
The course of microhardness in samples A1 to A10, (**a**) microhardness in the chrome layer, CHL = chrome layer; (**b**) microhardness in the substrate, S = substrate.

Figure 6 shows the results of the microhardness measurements in graphs for samples A1 to A10. These show the samples for each v_c_ and the smallest, middle, and largest v_f_ for both grinding wheels.

Based on all of the measurements carried out, it can be stated that the microhardness after grinding in all measured samples meets the requirements for this material, and there are no significant changes in the microhardness of the surface layer (in the same substrate). The obtained values correspond to a certain inhomogeneity of the samples, which is always expected. E.g. for sample A5, a decrease in microhardness to 925 HV0.1 can be observed in the chromium layer. This decrease in microhardness can be affected by a larger number and denser distribution of pores and cracks. The dynamics of microhardness in the surface layer of the base material are always very similar, when, just below the chromium layer, a slight increase in microhardness can be observed (when compared to the requirements for the base material, usually about 370 HV0.1). There is a slight decrease in microhardness to approx. 345 HV0.1, which meets the requirements for the base material. Substantially comparable results were measured for samples A6 to A10 (v_c_ = 40 m·s^−1^, SG).

**Figure 7:**
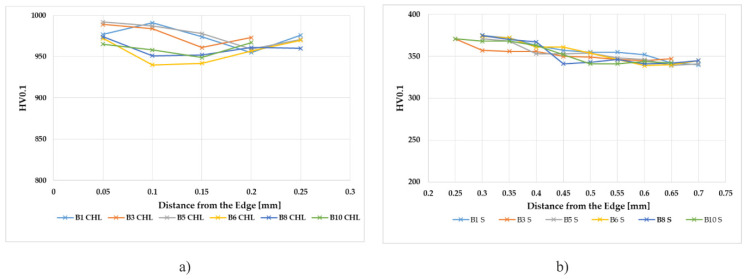
The course of microhardness in samples B1 to B10, (**a**) microhardness in the chrome layer, CHL = chrome layer; (**b**) microhardness in the substrate, S = substrate.

Similar results were also obtained for the grinding wheel SiC (samples B1 to B5, v_c_ = 30 m·s^−1^, B6 to B10, v_c_ = 40 m·s^−1^), see Figure 7.

### 3.3. Discussion

Based on the measurements that are described above, it was possible to make a few facts.

In terms of assessing the microstructure that was obtained after grinding, it can be observed that the experiment did not display any significant inherently good substrate microstructure changes when compared with the zero sample. After grinding, a slight increase in porosity could be observed in the microstructure. As expected, the microstructure of the source material identified sorbitol, corresponding measured values of microhardness.

From the point of view of the porosity evaluation, which is assumed to appear after grinding due to thermal and mechanical loading of the ground sample, it was possible to state for both grinding wheels used that increasing the feed rate v_f_ increased the load of samples and enlarged the pores and created new cracks, which was expected. However, these values were not significant compared to the uncut sample (porosity 0.03 ± 0.03%) and especially the milled samples. For samples ground SG, it was always possible to observe a slightly increasing porosity for both v_c_ with increasing v_f_. The smallest porosity was found in sample A1 and it amounted to 0.31 ± 0.10%. On the other hand, the highest porosity was achieved in sample A10 and it amounted to 2.60 ± 0.26%. In the case of crushed SiC specimens (B1 to B10), a lower overall porosity could be observed when the porosity limit of 2% is never exceeded. Samples B1 and B2 had essentially the same porosity (B1 0.59 ± 0.06%, B2 0.58 ± 0.07%), where the maximum porosity was shown by sample B5 (1.81 to 0.16%). The limit has not been exceeded for v_c_ = 40 m·s^−1^. Thus, we can say that the SiC wheel performed slightly better in this area, but the differences were noticeable, but small.

The evaluation of the microhardness of the applied chromium layer and substrate after grinding was the next evaluated aspect. The chromium layer after grinding has reached a thickness of 0.2 to 0.25 mm. The microhardness of the base material was measured in the surface layer at a depth of 0.4 mm. The measurement was carried out from the transition zone to the core of the sample. Looking for all of the microhardness courses in the chromium layer, it is seen that the values of microhardness to a depth of 0.05 mm after sanding were about 975 HV0.1. Along with the depth of the base material, the microhardness value increased from 350 HV0.1 (unloaded state, sample 0) to about 375 HV0.1 in the range from the transition zone to a depth of 0.15 mm. The microhardness value at a depth of 0.2 to 0.4 mm decreased to a value of the microhardness of the core material of approximately 350 HV0.1. After increasing the value of microhardness, a slight hardening occurred in the area below the transition region (between the chromium layer and the base material). The microhardness courses are approximately the same for both of the grinding wheels. Looking at the given cutting conditions, the microhardness values varied slightly, both in the chromium layer and in the base material. The experimental strengthening of the base material under the chromium layer should not affect the functionality of any component. The presented results suggest that there will be no delamination of the chromium layer after fracture under the presented cutting conditions. The courses of microhardness in the base material have a very similar character. Just below the chromium layer, the microhardness value reached approx. 370 HV0.1. Up to a depth of 0.05 to 0.1 mm, this value is at its maximum and gradually reaches a depth of 0.1 to 0.2 mm, and the value of microhardness is around 360 to 370 HV0.1. The microhardness value stabilizes at a depth of 0.4 mm from the base material at the core hardness (unreinforced material) by comparing all the microhardness courses in the surface layers, there was no significant change in microhardness as the cutting rate and feed rate changed. Only in the base material was the surface layer hardened to 0.2 mm after grinding. Changing the microhardness of the surface layer of the base material occurred from plastic deformation due to mechanical and thermal loads during grinding.

## 4. Conclusions

An experiment was performed under the conditions that are presented above and to a given extent. Furthermore, from measurements and analyses performed on this basis, we can state that the selected cutting conditions are acceptable from the point of view of the result of grinding.

Based on the realized measurements and analyses, in the case of the microstructure of the chromium layer and the base material, it can be stated:

- Before and after grinding, the structure of the base material was the same, i.e., it was retained. The initial microstructure of the base material was preserved and it was not changed in the transition area between the chromium layer and the base material.
- The structure of the base material was identified as sorbitol oriented along with the original martensitic needles.
- The reason is that the chromium layer could be affected and not show significant changes in the microstructure in the base material, which could potentially act as an insulator. It is likely that a transition area could be created if greater mechanical and thermal loads were applied, which evidently occurred under the given cutting conditions.
- From this point of view, suitable cutting conditions were presented.
- The evaluation of porosity in a layer of chromium may be summarized, as follows:
- A layer of chromium before the grinding process was analyzed with a porosity of 0.03%
- The porosity values after grinding ranged from 0.31% to 2.5%, which is acceptable in terms of internal conditions of the company, where the porosity is allowed up to 10%.
- The porosity value increased with the increasing feed rate. An increase in porosity also occurred when comparing the two cutting speeds of grinding wheels, where higher values were achieved for v_c_ = 40 m·s^−1^. Only in the comparison of samples B5 and B10 did the value of porosity decrease. This reduction manifested itself in grinding wheel SiC.
- Lower porosity values were achieved using grinding wheel SiC.
- While using the presented cutting conditions and grinding wheels, it is important to obtain very good porosity in the chromium layer.

It is possible to note the assessment of the microhardness of the surface layer and the base material:

- Microhardness in the chromium layer for both grinding wheels and under certain cutting conditions had a slightly different course of microhardness.
- The initial value of microhardness before the grinding process in the chromium layer was around 950 HV0.1.
- After grinding, the microhardness values of the chromium layer ranged between 960 to 975 HV0.1.
- The initial value of microhardness before the grinding process in the base material (substrate) was around 350 HV0.1.
- After grinding, the microhardness values in the base material just below the transition area increase to 365 to 385 HV0.1 and to a distance of 0.05 mm in the surface layer of the base material. At a depth of 0.05 to 0.2 mm of the base material, the microhardness values gradually decrease back to the core hardness value. Subsequently, at a depth of 0.2 to 0.4 mm, the microhardness values were around 350 HV0.1. Reinforcement in the surface layer of the base material occurred due to thermal and mechanical loading.
- These microhardness courses were similar for both of the grinding wheels.

In the values that are presented here, the grinding wheel SiC works a little better, albeit slightly. Therefore, it can be stated that this wheel achieved better results, although the difference between the two was very small and both types of grinding wheel were very well acceptable.

The selected cutting conditions and selected grinding wheels showed good results from the point of the microstructure view, which was not substantially affected, and the resulting porosity was small and well acceptable. This could be observed on all samples of the experiment.

Changes during the microhardness of the surface layer were small, and the technology used would depend on the result of grinding when cutting at predetermined conditions workload.

## Figures and Tables

**Table 1:** The chemical composition of the steel AMS6415 according to material list [34,35].

Chemical Composition [wt.%]								
C	Mn	P	Cr	Mo	S	Ni	Si	Fe
0.37–0.43	0.6–0.8	max. 0.035	0.7–0.9	0.2–0.3	max. 0.04	1.65–2.00	0.15–0.35	96

**Table 2:** Mechanical properties of steel AMS6415 [33,34].

Value	Min.	Max.
Tensile Strength in Tension Rm [MPa]	850	1555
Yield Strength in Tension Re [MPa]	635	1125
Ductility [%]	5	13
Hardness [HRC]	24	45

**Table 3:** Chemical composition of the substrate according to measurements (Q4 TAMAN).

Chemical Composition [wt.%]								
C	Mn	P	Cr	Mo	S	Ni	Si	Fe
0.415	0.711	<0.005	0.817	0.243	<0.001	1.835	0.263	95.300

**Table 4:** Cutting conditions of the experiment.

Material	Grinding Wheel	Cutting Conditions				Sample No.
		a_e_ [mm]	v_w_ [m·min^−1^]	v_c_ [m·s^−1^]	v_f_ [mm·min^−1^]	
**Galvanically applied chromium layer**	SG	0.05	15	30	0.13	A1
					0.17	A2
					0.26	A3
					0.41	A4
					0.64	A5
				40	0.13	A6
					0.17	A7
					0.26	A8
					0.41	A9
					0.64	A10
	SiC			30	0.13	B1
					0.17	B2
					0.26	B3
					0.41	B4
					0.64	B5
				40	0.13	B6
					0.17	B7
					0.26	B8
					0.41	B9
					0.64	B10

## Data Availability

Data available in a publicly accessible repository.

## Nomenclature

Symbols and acronyms	
AISI	American Iron and Steel Institute
CBN	Cubic Boron Nitrid
CCA	compressed cooled air
MQL	minimum quantity lubrication
SAE	Society of Automotive Engineers
SG	solgel, ceramic abrasives prepared by sol-gel method (microcrystalline corundum)
a_e_	removal depth [mm]
v_c_	cutting speed [m·s^−1^]
v_f_	radial feed of tool [mm·min^−1^]
v_w_	circumferential speed of the workpiece [m·min^−1^]
